# A window of opportunity: tear cytokine profiles in chronic ocular graft-versus-host disease

**DOI:** 10.1038/s41433-026-04674-z

**Published:** 2026-06-29

**Authors:** E. Greenan, F. O’Connell, E. Vandenberghe, E. Conneally, J. K. Dowling, S. L. Annett, J. O’Sullivan, J. Ní Gabhann-Dromgoole, C. C. Murphy

**Affiliations:** 1 Department of Ophthalmology, Royal Victoria Eye and Ear Hospital, Dublin, Ireland; 2 Department of Ophthalmology, RCSI, University of Medicine and Health Sciences, Dublin, Ireland; 3 School of Pharmacy and Biomolecular Sciences (PBS), RCSI, University of Medicine and Health Sciences, Dublin, Ireland; 4 Department of Surgery, Trinity St. James’s Cancer Institute and Trinity Translational Medicine Institute, St. James’s Hospital and Trinity College Dublin, Dublin, Ireland; 5 Department of Haematology, St James’s Hospital, Dublin, Ireland; 6 FutureNeuro, Research Ireland Centre, RCSI, University of Medicine and Health Sciences, Dublin, Ireland

**Keywords:** Corneal diseases, Biomarkers

Chronic ocular graft-versus-host disease (GvHD) complicates allogeneic stem cell transplantation, with immune-mediated inflammation and fibrosis of the ocular surface leading to severe dry eye disease (DED). While previous studies have demonstrated elevated tear cytokines in chronic ocular GvHD, few have characterised early-stage cytokine correlations with clinical severity [1–3].

The study was conducted in accordance with the Helsinki Declaration and received approval from the Research and Ethics Committee of St James Hospital (Ref: 2019-05 List 19). Consecutive patients with chronic ocular GvHD were recruited from a Haematology/Oncology clinic having met the National Institute of Health and/or the International Chronic Ocular GvHD Consensus Group (ICCGvHD) diagnostic criteria. Those on topical immunosuppressive agents or with pre-existing DED were excluded. Best Corrected Visual Acuity, Schirmer’s I test, tear break-up time (TBUT), Oxford surface staining (OSS), and ICCGvHD severity grading were assessed. Tear samples were collected, pooled and analysed using a 10-plex ELISA kit (Meso Scale Diagnostics, USA). Comparisons with matched healthy controls used Mann–Whitney tests and correlations used Spearman’s coefficient (*p* < 0.05), performed using GraphPad Prism 9.0.

Clinical and demographic characteristics are presented in Table 1. The cohort (8 male; mean age 43.9 ± 13.4 years) was representative of a chronic ocular GvHD population, with a mixed haematological diagnosis profile and spectrum of systemic GvHD. Participants had significant DED (mean Schirmer’s I: 4.2 ± 5.7 mm; TBUT: 3.8 ± 1.3 secs; OSS: 7.9 ± 4.2; mean 8.5 lubricant applications/day). ICCGvHD grading classified 53.8% as mild/moderate and 38.5% as severe. Nine of ten cytokines were significantly elevated versus controls (all except IL-4; Fig. 1A–J), confirming sustained immune-mediated inflammation in disease pathogenesis.

**Fig. 1:**
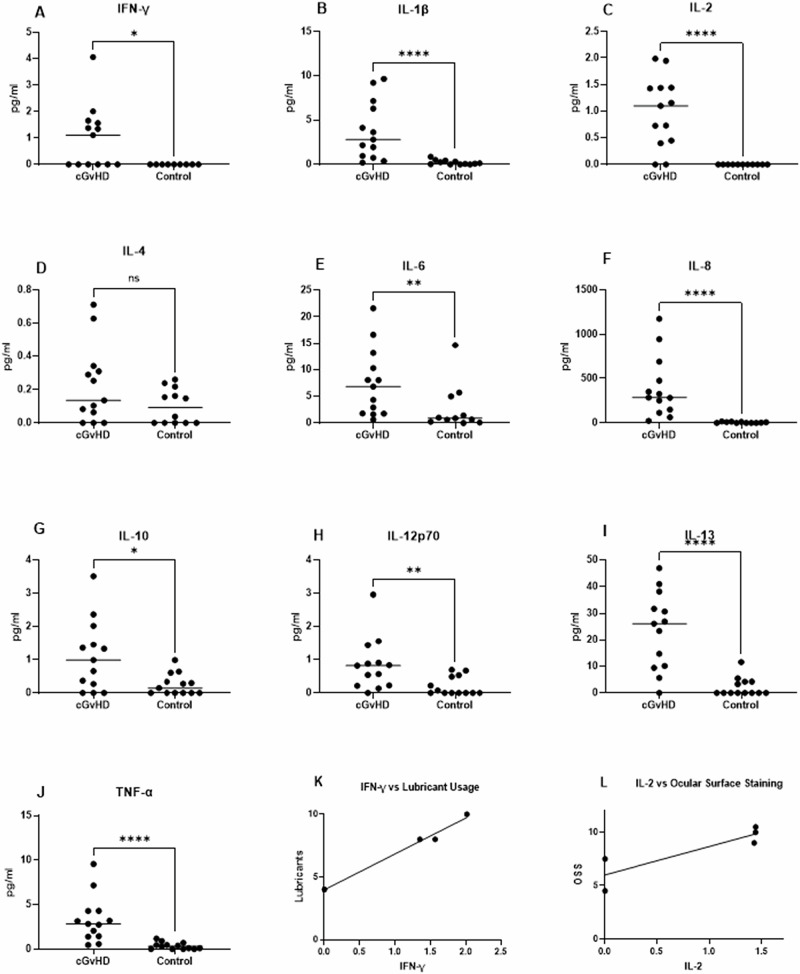
Ocular cytokine profiles in chronic ocular GvHD and their association with clinical disease parameters. **A–J** Cytokine levels from ocular washes of chronic ocular GvHD patients and health age- and gender-matched controls (*n* = 13), visually represented on scatter plots. Outliers were identified and removed prior to analysis (ROUT analysis, Q = 0.5%). Statistical analysis was assessed using Mann–Whitney test. ns denotes *p* > 0.05, *p ≤ 0.05, **p ≤ 0.01, ***p ≤ 0.001 and ****p ≤ 0.0001. **K–L** Scatterplots illustrating the correlative relationship between cytokines and clinical parameters. **K** IFN-γ versus frequency of lubricant usage recorded a correlation factor of 0.97, *p* = 0.03, **L** IL-2 versus ocular surface staining grades using the Oxford system, with a correlation factors of 0.98, *p* = 0.03. Statistical analysis was assessed using Spearman’s rank correlation coefficient.

**Table 1:** Clinical and demographic characteristics of the study cohort (*n* = 13).

		Mean	SD	Range	*n*	%
**Age (years)**		43.9	13.4	25.2–62.2	13	100
**Gender**	*Male*				8	61.5
	*Female*				5	38.5
**Diagnosis**	*AML*				7	53.8
	*HL*				2	15.4
	*Other*				4	30.8
**Type of allo-SCT**	*MUD*				8	61.5
	*Sib*				5	38.5
**Years since HSCT**		5.8	6.9	1.3–22.7	13	100
**Acute GvHD**	Skin				6	46.2
	Liver				4	30.8
	Other				4	30.8
**Chronic GvHD**	Ocular				13	100
	Skin				9	69.2
	Lung				3	23.1
	Oral				3	23.1
	Other				2	15.4
**Systemic immunomodulation**					8	61.5
**Ocular medications**	*Lubricant/ day*	8.5	4.6	4–20	13	100
	*ASD*				2	15.4
	*Ointment*				8	61.5
**BCVA**		0.1	0.1	-0.1 to 0.4	26	100
**OSS**		9.2	3.1	0–15	26	100
**Schirmer I test (mm)**		5.0	6.4	0–21	26	100
**TBUT (sec)**		3.2	1.2	1–5	13	100
**MGD**					8	30.8
**Disease Severity**						
*ICCGvHD*	*None*				1	7.7
	*Mild/ Moderate*				14	53.8
	*Severe*				10	38.5
*NIH*	*Score 1*				0	0
	*Score 2*				18	69.2
	*Score 3*				8	30.8

*AML* acute myeloid leukaemia, *ASD* autologous serum drops, *BCVA* best-corrected visual acuity, *HL* Hodgkin lymphoma, *MUD* matched unrelated donor, *Sib* sibling donor, *GI* gastrointestinal, *GvHD* graft-versus-host disease, *HSCT* haematopoietic stem cell transplantation, *ICCGvHD* International Chronic GvHD severity score, *MGD* meibomian gland dysfunction, *MUD* matched unrelated donor, *NIH* National Institutes of Health, *OSS* ocular surface staining, *Sib* sibling donor, *TBUT* tear breakup time.

In patients with early-stage disease (<1year duration; *n* = 5), IFN-γ correlated strongly with lubricant usage frequency (*r* = 0.97, *p* = 0.03; Fig. 1K) and IL-2 with OSS severity (*r* = 0.98, *p* = 0.03; Fig. 1L), suggesting that these mediators are most active during disease establishment rather than in the later chronic, fibrotic stage. IFN-γ is a key Th1 effector cytokine implicated in goblet cell loss, reduced mucin production, and conjunctival epithelial apoptosis [1, 3, 4]. IL-2 drives effector T-cell proliferation and Th1/Th2 differentiation and its correlation with surface damage suggests that its pro-inflammatory activity outweighs its regulatory T-cell–maintaining function in early disease [5].

These findings identify a stage-specific inflammatory signature in early chronic ocular GvHD, with IFN-γ and IL-2 emerging as clinically actionable targets. This indicates that early cytokine-directed intervention, rather than tear replacement alone, may represent a tractable strategy to interrupt disease progression before irreversible fibrotic damage occurs on the ocular surface.

This study is limited by a small sample size. However, differing from previous studies where referral bias may skew cohorts towards more severe disease, consecutive recruitment from a national transplant centre allowed for a more representative capture of chronic ocular GvHD [2, 3]. Strict exclusion criteria also reduced confounding, and strong correlations and biological plausibility support the findings.

## Data Availability

The study data is not available, as participants did not provide consent for data sharing at the time of the study.
